# Stochastic and deterministic multiscale models for systems biology: an auxin-transport case study

**DOI:** 10.1186/1752-0509-4-34

**Published:** 2010-03-26

**Authors:** Jamie Twycross, Leah R Band, Malcolm J Bennett, John R King, Natalio Krasnogor

**Affiliations:** 1 Centre for Plant Integrative Biology, School of Biosciences, Sutton Bonington Campus, University of Nottingham, Nottingham, LE12 5RD, UK; 2 School of Mathematical Sciences, University Park, University of Nottingham, Nottingham, NG7 2RD, UK; 3 Automatic Scheduling and Planning Group, School of Computer Science, Jubilee Campus, University of Nottingham, Nottingham, NG8 1BB, UK

## Abstract

**Background:**

Stochastic and asymptotic methods are powerful tools in developing multiscale systems biology models; however, little has been done in this context to compare the efficacy of these methods. The majority of current systems biology modelling research, including that of auxin transport, uses numerical simulations to study the behaviour of large systems of deterministic ordinary differential equations, with little consideration of alternative modelling frameworks.

**Results:**

In this case study, we solve an auxin-transport model using analytical methods, deterministic numerical simulations and stochastic numerical simulations. Although the three approaches in general predict the same behaviour, the approaches provide different information that we use to gain distinct insights into the modelled biological system. We show in particular that the analytical approach readily provides straightforward mathematical expressions for the concentrations and transport speeds, while the stochastic simulations naturally provide information on the variability of the system.

**Conclusions:**

Our study provides a constructive comparison which highlights the advantages and disadvantages of each of the considered modelling approaches. This will prove helpful to researchers when weighing up which modelling approach to select. In addition, the paper goes some way to bridging the gap between these approaches, which in the future we hope will lead to integrative hybrid models.

## Background

Biological systems are naturally multiscale and to understand their behaviour fully we must understand the interaction of a number of processes that may occur on diverse temporal and spatial scales. To gain insight into such multiprocess and multiscale systems, there is a range of modelling frameworks that could potentially be employed. Different modelling approaches serve to highlight certain aspects of a biological system, and which modelling approach is most appropriate depends on the biological questions that are being addressed, as well as on the available data that could be used to calibrate or validate a given model. In this paper, we present several modelling approaches and show how these can be used to gain understanding of a realistic multiscale systems biology problem. We compare the different modelling approaches to each other and discuss their applicability.

To compare the modelling approaches, we focus on a particular case study: the transport of the hormone auxin through a file of plant cells. Auxin plays a major role in many aspects of plant growth and development [1]. It moves through the plant in a polar manner due to non-uniform spatial distributions of active influx and efflux carriers on the cell membranes [2], and the resulting auxin distributions influence a wide range of processes, including organ initiation [3-6], vein formation [7-12] and gravitropism [13]. Modelling auxin transport is thus an active research area in plant systems biology. The models are inherently multiscale, as cell-scale processes lead to tissue-scale phenomena. To date, the majority of modelling in this area computes solutions by simulating large systems of deterministic ordinary differential equations [2-9,11-17], and there are relatively few examples of alternative modelling techniques [18-20]. This paper complements such previous work by highlighting the benefits of using multiple modelling techniques to gain a more comprehensive understanding of a biological system, in this case auxin transport in plant tissue.

Mathematical modelling is routinely used to study biological phenomena quantitatively, often by describing the dominant physical processes using systems of coupled differential equations and solving these governing equations using analytical and numerical methods; such techniques have been used to study a diverse range of biological processes, including population dynamics, pattern formation, neuron firing and physiological flows (see [21] and [22] and references therein). Mathematical models have denotational semantics in that they represent relationships between quantities as systems of equations. In contrast, computational, or executable, models have operational semantics, and define rules that describe how the modelled system moves from one state to the next [23]. Computational models are executed in the sense that, starting from an initial state, a procedure (in our case a stochastic simulation algorithm) determines the next reaction to apply. This reaction is then applied, giving a new system state, which is then used by the simulation algorithm to determine the next rule to apply, in an iterative procedure. Stochastic processes, unlike their deterministic counterparts, involve an indeterminancy in the evolution of the state of the system. For large numbers of molecules, this stochasticity may be averaged out, giving what appears to be a deterministic process; however, when a small number of molecules is involved, stochastic effects become evident, and in such cases the system may behave in a markedly different way. The inherent noise present in all biological systems is explicitly modelled in discrete stochastic models and can have profound effects on system dynamics, producing behaviour, even for large numbers of molecules, which is markedly different from that predicted by continuous deterministic models; see, for example, [24].

After summarising the biological abstraction which forms a common basis for the models presented in this paper, we describe a stochastic computational model based around a P system framework. Here the number of auxin molecules in each compartment evolves according to rules that move molecules from one compartment to the next. We then describe a deterministic mathematical model in which the auxin concentrations are described by a system of coupled ordinary differential equations. In the deterministic case, we produce two solutions: i) analytical solutions, derived using multiscale asymptotic approaches, and ii) numerical solutions, as is typical in current auxin-transport modelling.

## Methods

### Biological Abstraction

To investigate the benefits of deterministic and stochastic modelling approaches, we focus on a model of auxin transport. Specifically, we model a standard experiment that is used to determine auxin velocities: radio-labelled auxin is added to a source agar block at one end of a segment of stem tissue, auxin then travels through the stem segment, and experimentalists measure the amount of auxin collected in a final agar block at the other end of the stem segment (see [25-27] and references therein).

**Figure 1:**
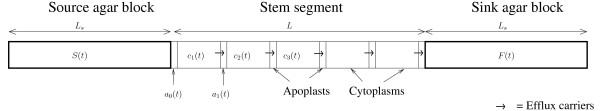
**Model of a single file of cells**. In the model, auxin molecules are initially in the source agar block, travel through the stem segment and are collected in the final agar block. We model the stem segment as a single two-dimensional line of *N *cells for simplicity, and suppose that efflux carriers are located on the downstream face of each cell membrane. We solve for the auxin concentrations in the two agar blocks, the *N *cytoplasm compartments and the *N *+ 1 apoplast compartments.

As shown in Figure 1, we model the stem segment as a single two-dimensional line of *N *cells, where each cell contains a cytoplasm (with length *l *and width *w*), and there is a layer of apoplast between neighbouring cytoplasms (which has thickness *λ *and width *w*). Hence the total length of the stem segment is *L *= *Nl *+ (*N *+ 1)*λ*. The model assumes that the auxin concentration within each compartment is uniform, which is a reasonable assumption given the small size of the compartments and the rapidity of auxin diffusion.

We suppose that the two agar blocks are rectangular, with length *L*_*s *_and width *w*. We denote the number of auxin molecules in the source agar block by *S*^*n*^(*t*) and the number in the collecting agar block by *F*^*n*^(*t*), where the superscript *n *emphasises that these quantities are numbers of molecules; the concentrations (i.e. number per unit area) in the two agar blocks are then given by *S*(*t*) = *S*^*n*^(*t*)/(*L*_*s*_*w*) and *F*(*t*) = *F*^*n*^(*t*)/(*L*_*s*_*w*), respectively. We suppose that auxin diffuses within the agar blocks with diffusion coefficient *D*. Auxin in the source agar block diffuses into the adjacent root apoplast region; it then travels through the line of *N *cells and diffuses from the final apoplast region into the collecting agar block. We denote the number of molecules in the cytoplasm by  for *i *= 1, 2, ..., *N*, and the number in the adjacent apoplast by  for *j *= 0, 1, 2, ... *N*, with the corresponding auxin concentrations given by *c*_*i*_(*t*) = /(*lw*) and *a*_*i*_(*t*) = /(*λw*) (see Figure 1).

Auxin exists *in planta *in either an anionic or a protonated form. Following previous auxin-transport models, we assume that auxin molecules rapidly associate or dissociate, so that the proportion of these two forms are in equilibrium and are determined by the *pH *of the region in which it is located and by the auxin dissociation constant, *pK*. Using the subscripts *c *and *a *to refer to the cytoplasm and apoplast respectively, the fractions of protonated and anionic auxin are given by [14](1)

In the line of cells, auxin moves between the apoplast and cytoplasms by crossing the cell membrane. The flux of protonated auxin across the membrane, *J*_*diff*_, is passive, whereas the flux of anionic auxin is mediated by PIN efflux carriers that are present on the downstream face of each cell membrane (see Figure 1); following [13], we model the anionic flux across the cell membrane, *J*_*PIN*_, using Goldman-Hodgkin-Katz theory (see [21] for details). Thus, the components of the flux from the cytoplasm *c*_*i *_to the apoplast *a*_*i *_are given by(3)

where(4)

and *P*_*diff *_is the membrane permeability of protonated auxin, *P*_*PIN *_is the membrane permeability of anionic auxin, and the dimensionless constant *ϕ *≡ -*F*_*D*_*V*/*RT *where *F*_*D *_is the Faraday constant, *V *is the membrane potential, *R *is the gas constant, and *T *is the temperature.

At time *t *= 0, all the auxin molecules are in the source agar block; we prescribe *S*^*n*^(0) = *C*, and let  =  = *F*^*n*^(0) = 0 for *i *= 1, 2, ... *N *and *j *= 0, 1, 2, ... *N*. We model a closed system, that is, we assume that, during the subsequent auxin movement, no auxin molecules enter or leave the system. Table 1 summarises estimates of the model parameters based on values reported in the biological literature; these are discussed further in the "Biological Parameter Estimates" section of Additional file 1.

**Table 1:** Biological parameter estimates.

Parameter	Description	Value
*l*	cytoplasm length	100 *μ*m

*w*	cytoplasm width	10 *μ*m

*L*	tissue length	2 × 10^-3 ^m

*L*_*s*_	agar-block length	2 × 10^-3 ^m

*λ*	apoplast thickness	0.5 *μ*m

*P*_*diff*_	membrane permeability	5.6 × 10^-7 ^m s^-1^

*P*_*PIN*_	PIN permeability	3.3 × 10^-6 ^m s^-1^

*D*	diffusion coefficient	6.7 × 10^-10 ^m^2 ^s^-1^

*pH*_*c*_	cytoplasm pH	7.2

*pH*_*a*_	apoplast pH	5.3

*pK*	dissociation constant	4.8

*V*	cell membrane voltage	-0.120 V

*T*	temperature	295.15 K

*F*_*D*_	Faraday constant	96485.3399 Cmol^-1^

*R*	gas constant	8.314472 K^-1 ^mol^-1^

Parameters used for both the stochastic and deterministic models.

### Stochastic Computational Model

To obtain stochastic solutions, we use a multi-compartment stochastic P system framework [28]. Individual molecules of auxin are modelled as objects which move between compartments according to a set of rules associated with each compartment. Compartments of the same type have the same set of rules associated with them, and each rule has an associated stochastic reaction constant *k *which determines the rate at which the rule transports molecules (see Table 2). We define rules for auxin diffusion between source/collecting blocks and the apoplast, membrane diffusion from apoplast to cytoplasm, and carrier-mediated efflux from cytoplasm to apoplast. These reaction constants are related to the parameters given in Table 1 via(5)

**Table 2:** Stochastic model rules.

ID	rule	process
**source agar block **(*S*)		

*R*_1_		diffusion

**collecting agar block **(*F*)		

*R*_2_		diffusion

**cytoplasms **(*c*_*i*_)		

*R*_3_		PIN transport

**apoplasts **(*a*_*i*_)		

*R*_4_		membrane diffusion

*R*_5_		membrane diffusion

*R*_6_		PIN transport

*R*_7_		diffusion

*R*_8_		diffusion

Molecules *A *of auxin are transported from compartment *i *to a neighbouring compartment at rates *k *which depend on the type of transport process. For apoplasts, rules *R*_4 _and *R*_5 _are assigned to apoplasts bordered on both sides by cytoplasms. Rule *R*_7 _replaces rule *R*_5 _for the first apoplast, and *R*_8 _replaces *R*_4 _for the final apoplast, which are bordered by the source and collecting agar block respectively.

(further information and derivations can be found in the "Model Derivation" section of Additional file 1). We note that in this framework we set the small parameter  to zero to enable efficient execution. The computational model is executed using a novel multi-compartment Monte Carlo stochastic algorithm using the mcss simulator, which is part of the Infobiotics workbench, a freely available software suite for designing, simulating and analysing multiscale executable systems and synthetic biology models [29]. Stochastic algorithms and software supporting multiple compartments have been developed by several research groups [30-33]. A key difference between these algorithms and our software suite is that our tools support the rapid prototyping of models by facilitating the abstraction of commonly occurring motifs (e.g. regulatory or signalling motifs [34]) with model templates and modules. This, coupled with the facility to explicitly specify a tissue geometry, permits the seamless exploration of "*what if *" scenarios during model building. For example, to reproduce the behaviour of a mutant which does not produce a particular protein, all that needs to be done is to set the rate constant of the reaction producing the protein to zero. Or, for example, to remove a particular regulatory mechanism, we can simply remove the module representing this regulatory mechanism from the list of modules employed by a particular compartment. The reader is referred to [28] for an in-depth description of the stochastic simulation algorithm we employ, which we briefly summarise here. Essentially, the simulation algorithm determines, within each of the simulated compartments, which rule to apply next and when to apply it. This highlights the key difference between mathematical and computational (or algorithmic) models - mathematical equations describe the change in the values of variable as a system moves from one state to the next, while computational models expose how and why this state change occurs [23,35]. Each run of the simulation gives one possible trajectory of the model through state space. Hence, as well as using individual runs to examine the reasons for state changes, we can execute the model a number of times to estimate the average system behaviour, analyse the system's variability, and identify potential extreme behaviours.

Additionally, the computational model enables model checking, the formal verification of model behaviour, which allows the identification of general biological principles which underlie the observed behaviour of the model [36-39]. Using algorithms such as those presented in [40], the computational model also facilitates model parameter and structural optimisation, allowing incomplete biological information regarding, for example, model parameter settings and structure, to be established automatically.

### Deterministic Mathematical Model

In the deterministic framework, the auxin concentration in each region is governed by the following system of equations(6)

for *i *= 1, 2, ..., *N *and *j *= 1, 2, ..., *N *- 1. The dynamics described by (6-11) are analogous to the reactions in (5), as shown in the "Model derivations" section of Additional file 1. We assume that initially all the auxin molecules are in the source agar block, therefore *S *= *C*/*L*_*s*_*w*, *c*_*i *_= *a*_*j *_= *F *= 0 at *t *= 0 for *i *= 1, 2, ... *N *and *j *= 0, 1, 2, ... *N*.

We now use asymptotic methods to derive an analytical solution for these governing equations, (6-11): these explicitly contain at least two distinct length scales, namely the cell length scale, *l*, and the (much larger) tissue length scale, *L *= *Nl *+ (*N *+ 1)*λ*, - it is this multiscale aspect that we exploit in deriving a continuum formulation. For comparison, we also solve the deterministic governing equations numerically using Matlab's ordinary differential equation solver, *ode45*. To obtain an analytical solution, we consider the dynamics on the time scale of active transport the length of the tissue, *L*/*P*_*PIN*_, and nondimensionalise using(12)

The model then depends on the dimensionless parameters(13)

To make analytical progress, we construct an asymptotic solution in the biologically appropriate limit in which ϵ is small, i.e. the length scale *L *of the tissue is much larger than that of a single cell, *l *+ *λ*, expanding the concentrations as standard perturbation series. Based on the biologically relevant parameter estimates in Table 1, we assume that the relative length of the agar blocks,  = *O*(1) as ϵ → 0^+^, and rescale the small parameters(14)

where  and  are *O*(1) as ϵ → 0^+^. In the stem segment, the result is a continuum limit. Letting *x *measure the length along the tissue, such that *x *= ϵ*i*, we consider *c*_*i*_(*t*) = *c*(*x*, *t*), *a*_*i*_(*t*) = *a*(*x*, *t*), where *x *= 0 corresponds to the upper face of cytoplasm *i *= 1.

Based on these assumptions, we can derive formulae for the leading-order auxin concentration in each compartment (see the "Derivation of Asymptotic Solution" section of Additional file 1 for details). We identify two time scales. On the transport time scale,  = *O*(1), the source-agar-block concentration does not deplete significantly,  ≈ 1, and auxin travels through the stem segment with a defined front according to a wave equation, with effective velocity(15)

The sink-agar-block concentration is small, and given by(16)

On a longer time scale, , the cytoplasm concentrations in the stem segment are uniform, and the agar-block concentrations are given by(17)

therefore, diffusion within the agar blocks determines the rates at which the source-agar-block concentration depletes and the sink-agar-block concentration increases.

## Results and Discussion

### Auxin Concentrations

**Figure 2:**
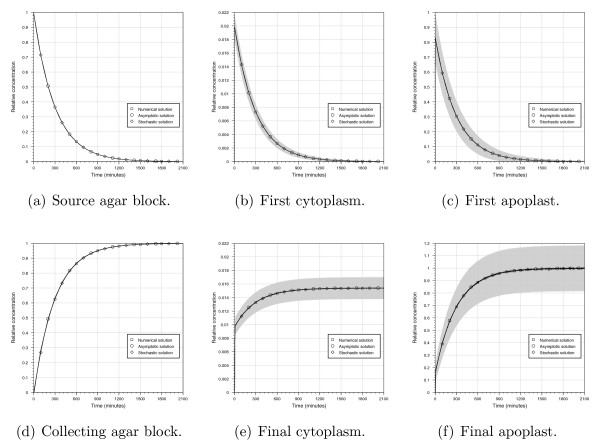
**Predicted auxin concentrations from the deterministic numerical, deterministic asymptotic and stochastic model solutions**. Concentrations are given relative to the initial source concentration *C*/(*L*_*s*_*w*). For the stochastic solutions, the mean concentrations are calculated over 10,000 runs, and 95% confidence intervals are given as grey ranges.

For both the deterministic and stochastic frameworks, the relative auxin concentrations in the source agar block, collecting agar block, cytoplasm and apoplast regions are shown in Figure 2. For the stochastic model, we show both the mean concentrations and the 95% confidence intervals (which are calculated using 10,000 runs). To compare quantitatively the solutions of the deterministic and stochastic models, we compute the time taken for the agar-block concentrations to reach half their steady-state levels. These results, given in Table 3 and Figure 2, demonstrate excellent agreement between the three modelling approaches.

**Table 3:** Stochastic and deterministic model steady-state concentration times.

model	source (min)	sink (min)
**deterministic (numerical)**	206.06	213.13

**deterministic (analytical)**	206.21	206.21

**stochastic**	206.28 ± 2.88	213.63 ± 2.88

Time taken to reach half the steady-state concentrations for the deterministic and stochastic models. 95% confidence intervals are also given for the stochastic model.

**Figure 3:**
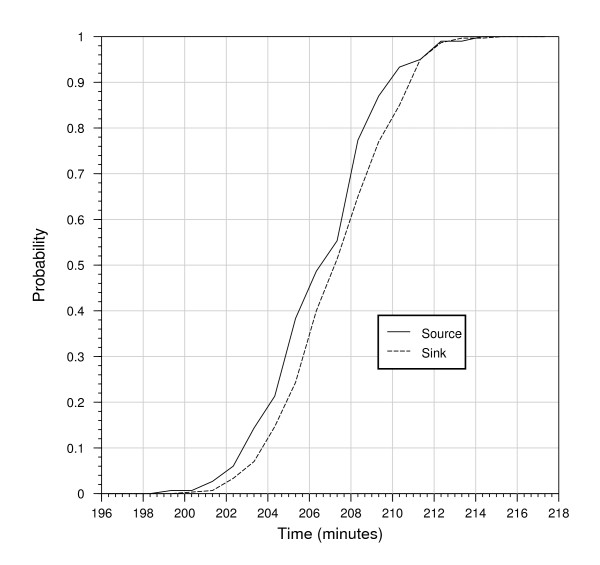
**Model checking results for the stochastic computational model**. The abscissae show simulation time and the ordinates the probability that half the steady-state concentration is reached.

**Figure 4:**
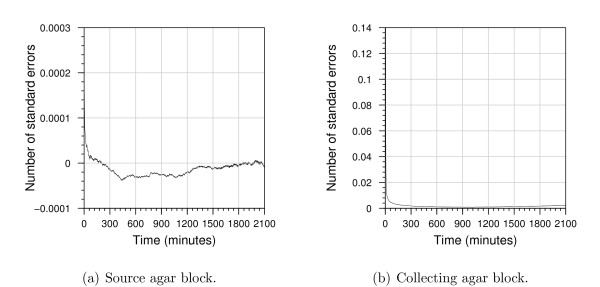
**Differences between stochastic solutions and analytical solutions of the deterministic model**. Each plot shows the number of standard errors by which the concentration predicted by the deterministic model differs from the mean concentrations predicted by the stochastic model over 10,000 runs.

Computational models, because of their algorithmic specification, are amenable to model checking. Model checking allows formal verification that a model satisfies a prescribed property. Properties are propositions about the state of the model, for example, the amount of species *A *that reaches a level *x*. For the computational model, we used the Infobiotics workbench to perform probabilistic model checking and formally determine the probability of the agar-block concentrations reaching half their steady-state levels. Due to computational constraints, we check a reduced version of the computational model (see the "Model Checking" section of Additional file 1 for details). Figure 3 shows that, with 95% confidence, after 215 minutes both the agar-block concentrations will have reached half their steady state, and that the source-agar-block will reach its steady-state concentration slightly before the collecting agar block. To characterise when the deterministic and stochastic models agree and differ, we subtract the concentration predicted by the analytical deterministic model from the mean concentration predicted by the stochastic model and divide this value by the standard error given by the stochastic model; the predicted concentrations are then considered to differ significantly if the absolute value of the result is greater than two, that is, if the difference between the two predicted concentrations is greater than two standard errors. Figure 4 shows that there is no significant difference between the source concentrations predicted by the deterministic and stochastic models. In contrast, in the collecting agar block there is a significant difference for the initial 196 seconds, with a maximum difference of six standard errors. The deterministic approach takes the concentrations to be continuous while the stochastic model considers individual molecules. As a result, for the first 138 seconds, the stochastic model typically predicts a zero concentration, whereas the deterministic model predicts a small positive concentration. As shown in Figure 4, for the remaining time, although not significantly different, the stochastic model consistently predicts larger concentrations than the deterministic one.

To assess how the initial source concentration affects the variability of the auxin concentrations, we performed 10,000 runs of the stochastic computational model with initial concentrations of 0.1 nM and 1 nM (see Table 4). The mean variability and its standard deviation in all compartment types decreases by around 69% as the initial concentration is increased from 0.1 nM to 1 nM. Theoretically, the amount of noise in a stochastic simulation is of the order of , where *n *is the number of molecules [41]. Since  ≈ 0.31, we would theoretically expect a 69% decrease in noise, in agreement with the simulation results.

**Table 4:** Stochastic model concentration variability.

	initial concentration	
	
	0.1 nM	1 nM
**source**	0.00184 ± 0.00137	0.00059 ± 0.00043

**sink**	0.00187 ± 0.00135	0.00059 ± 0.00043

**cytoplasm**	0.00006 ± 0.00006	0.00002 ± 0.00001

**apoplast**	0.00003 ± 0.00003	0.00001 ± 0.00001

Variability of concentrations observed in the stochastic computational model for different initial concentrations. Each column gives the mean and standard deviation of the standard deviation of auxin concentrations observed.

### Model applicability

Selecting the appropriate modelling approach for a given problem involves a number of factors including a researcher's judgement of the time available for model development and the computational resources available. A key factor is the understanding they wish to obtain, for example, arriving at the best possible model for a given system (in which case model development time might not be too important), or generating alternative plausible models representing competing experimental hypothesis (in which case the availability of rapid prototyping, averages and outlier behaviours are needed). Although difficult to give all-encompassing guidelines, in this section we briefly discuss general practical considerations arising from our case study that are involved in determining an appropriate modelling approach.

The stochastic computational model needs to be executed many times to assess the average behaviour of the system for a given set of parameter values, and therefore the computational cost of solving the stochastic model will generally be greater than that of the deterministic model. The execution time of the computational model depends on the values assigned to the reaction constants and the initial numbers of molecules present in the system (as with more molecules there are more reactions per second). Table 5 summarises the time taken to execute the computational model for several initial concentrations, and compares these execution times with the time taken to solve the deterministic numerical model. Tests were performed on a single processor (AMD Athlon 64 X2 Dual Core 5600+ 2.8 GHz with 1 GB of memory) - using multiple processors would reduce these execution times. As shown in Table 5, as expected, the execution time of the stochastic model increases as the initial number of molecules increases. For initial auxin concentration above 1 nM, the stochastic model will take a considerable amount of time, and therefore in this case it would be advisable to use a deterministic approach. In contrast, the asymptotic solution of the mathematical model does not involve any computational cost (other than the negligible time required to evaluate the derived expressions at a given timepoint). However, the computational execution time does not reflect the true time taken to obtain the solution, as deriving the asymptotic formulae may take longer than producing the numerical or stochastic models.

**Table 5:** Stochastic and deterministic model execution times.

model	execution time
**deterministic (numerical)**	0.19 seconds

**stochastic **(*C *= 10 pM)	1.19 hours

**stochastic **(*C *= 0.1 nM)	6.63 hours

**stochastic **(*C *= 1 nM)	62.30 hours

For the stochastic model, the execution time is the time required to perform 10,000 runs and depends on the total number of molecules present (which is determined by the initial auxin concentration).

When considering the time costs of different modelling approaches, a key consideration is the number of different parameter choices one wishes to investigate. The formulae from the analytical approach clearly show how the parameter estimates affect the predicted concentrations and transport speeds provided the scaling, (14), holds. However, we would need to derive a new asymptotic solution if we wanted to consider different parameter regimes. In addition, the asymptotic method presented here is can only be applied if we can consider the tissue to be a continuum, which is only appropriate if the rate of transport between the cells is not too small [21]. Using the numerical deterministic and stochastic approaches, one can use any parameter values in the simulations by making simple changes to the numerical code without changes to the underlying model. However, using these methods, we would need to execute many simulations to thoroughly understand how the dynamics are affected by the parameter values, which needs to be balanced with the increased execution time for the stochastic model for certain parameter regimes.

In summary, the asymptotic model is applicable only to the specific parameter regime for which it was derived, but allows rapid evaluation of the behaviour of the model within these bounds. However, to explore model behaviour outside of the given parameter regime, the asymptotic solution will need to be derived anew. The stochastic computational model allows any parameter regime or spatial scaling to be explored without further reformulation of the model and formally captures the *mechanisms *involved in producing a given phenotype. For some regimes the execution time of the model will be considerable, although this time can be ameliorated through the application of more computational resources or parallel computation.

### Auxin-Transport Speed

Auxin-transport experiments aim to investigate the movement of auxin through plant tissue. We have modelled an experimental protocol, as described in [25,27], that has been used to consider both the distance moved by auxin molecules per unit time (the velocity) and the amount of auxin passing through the tissue per unit time (the flux). In the deterministic model, auxin from the source agar block moves through the tissue with a defined front, and the asymptotic solutions provide a simple formula for the speed of transport (15). However, in practice there will be stochasticity in the auxin movement. The stochastic model predicts when the first auxin molecule appears in the collecting agar block, and the transport speed can be calculated by dividing the total length of the stem segment, *L*, by the average time taken for the first molecule of auxin to appear in the collecting agar block (this time is calculated by averaging over 10,000 runs).

The results, summarised in Table 6, show that the stochastic approach predicts a greater auxin-transport speed (3.38 ± 0.3 cm·h^-1^) than the asymptotic deterministic solution (1.95 cm·h^-1^). We note that this discrepancy is in agreement with the discussions in "Auxin Concentrations" section above where we showed that the different modelling frameworks predicted significantly different concentrations in the collecting agar block at early times.

**Table 6:** Stochastic and deterministic model transport speeds.

model	time to collecting agar block (s)	velocity (cm·h^-1^)
**deterministic**	356.91	1.95

**stochastic**	213.85 ± 21.18	3.38 ± 0.30

Auxin-transport speeds for the deterministic (asymptotic) and stochastic models. 95% confidence intervals are also given for the stochastic model.

Auxin velocities are generally thought to be around 1 cm·h^-1^, which is fairly close to our predictions, given that the velocity depends on the parameter estimate for *P*_*PIN *_and this value is not well characterised. One reason for the difference between the experimental auxin velocity and the model predictions may be differences in auxin detection sensitivity between the wet experiments and models. The stochastic model enables us to predict the time at which the first molecule of auxin enters the collecting agar block. However, in the wet experiments, a certain amount of auxin must accrue in this agar block before detection is possible. We can estimate the amount of auxin present in the collecting block from our models. If we consider an experiment with 12, 044 molecules of auxin and assume that, in line with experimental results, the auxin-transport speed is 1 cm·h^-1^, then the time taken for auxin to travel the length of the stem segment is 723.78 s (0.20105 hr). The deterministic model gives the number of molecules in the collecting agar block at this time to be 191 and 246 from the numerical and asymptotic solutions respectively (we note that this accuracy is within the expected range for the asymptotic solution). The mean number of auxin molecules at this time in the collecting agar block calculated over 10,000 runs of the stochastic model is 299.40 ± 15.84. Thus, to determine accurately the presence of auxin in the collecting agar block, the experimental apparatus used must have a sensitivity of 1.6 pM for an agar block of the same size as we simulated, and a finer resolution for larger agar blocks.

As discussed in [27], the majority of auxin-transport measurements report the flux of auxin transport rather than the auxin velocity and so consider the amount of auxin that has moved through a specified distance of tissue in a constant amount of time. However, the asymptotic solutions of the deterministic model demonstrate that diffusion within the agar blocks may significantly affect the auxin concentration within the collecting agar block, and therefore the auxin fluxes measured. The analysis presented in the "Derivation of Asymptotic Solution" section of Additional file 1 shows that there are two disparate time scales: on a short time scale, auxin is transported through the stem segment, whereas over longer ones, the auxin concentrations are almost uniform throughout the stem segment, and the dynamics are dominated by diffusion within the agar. It is clearly important to be aware of these two processes when interpreting experimental results. If an auxin-transport experiment were carried out over several hours, the auxin concentration in the collecting agar block would be determined predominantly by the diffusion rate. We emphasise that these conclusions are based on the assumption that the agar-block length is comparable to the stem-segment length - the effect of diffusion within the agar blocks will be less significant with smaller agar blocks.

## Conclusions

In systems biology, models are typically deterministic and a biological problem is translated into large systems of ordinary differential equations that are solved numerically. However, this is not the only option, and in this paper we have demonstrated three different modelling approaches: (i) deterministic numerical; (ii) deterministic asymptotic; and (iii) stochastic computational. As expected, particularly given that the dynamics can be described by a system of linear governing equations, there is excellent agreement between the three methods.

We have focussed our case study on auxin transport, as this is inherently multiscale with cell-scale dynamics creating the tissue-scale phenomena of interest. The numerical, deterministic method focusses on computing the cell-scale dynamics, whereas the asymptotic method makes use of the multiscale nature of the system: in the asymptotic results, we consider the auxin concentrations on the cell scale, and exploit the relatively small dimensions of the cells to determine how the cell-scale dynamics lead to effective tissue-scale behaviour. The stochastic computational model simulates the interaction of auxin at a molecular scale and, by analysing the gross movement of auxin from one compartment to the next, allows us to determine auxin dynamics at the tissue scale based on the *mechanistic *interactions of auxin at the molecular scale.

The model results enable us to highlight the advantages of each approach. We solved the stochastic version of the model using a P system framework: the model is written in terms of numbers of molecules and we prescribe the probability of a molecule moving between compartments. P systems are highly intuitive, and an excellent way of engaging with a biological audience. The stochastic model generates in particular both the mean and the standard deviation of the auxin concentrations, which enables us to characterise the expected variability. We also solved the model by deriving deterministic ordinary differential equations and using asymptotic methods to obtain formulae for the auxin concentrations and transport speeds. This method requires careful analysis to determine the dominant processes on each time scale, and the resulting expressions show clearly how the model parameters affect the predicted auxin concentrations and speeds. Although numerically solving the deterministic version of the model is often the quickest method of producing a solution, stochastic P system models and asymptotic analysis can provide additional insight and information that can complement, or be an alternative, to a deterministic numerical solution. The results also highlight how the experimental set up may lead to potential discrepancies between the measured auxin velocities, and, in particular, how the measured velocities will be affected by diffusion within the agar block. Auxin speeds are generally assessed by measuring the number of auxin molecules in the collecting agar block; however, we showed that on long time scales the auxin concentration in the agar block depends on the agar-block length, and the formulae for the auxin velocity and collecting-block concentration (obtained from the asymptotic analysis) are clearly not related. We could gain further understanding of the biological implications of this result by extending the model to incorporate a more accurate representation of the stem segment, for example by modelling multiple cell files with tissue-specific active transport.

## Authors' contributions

JT and LRB conceived and designed the models and experiments, performed the experiments, analysed the results, and drafted the manuscript. JT wrote the software to simulate the stochastic model and produced the stochastic solutions, whereas LRB produced the deterministic solutions. NK and JRK contributed to the conception and design of the models and experiments, and analysis of the results, and NK, JRK and MJB helped draft the manuscript and attracted funds for carrying out this work. All authors read and approved the final manuscript.

## Supplementary Material

Additional file 1 **Further model details**. This document (PDF format) provides supporting information for the main text, and gives further details on the biological parameter estimates used in the model; the derivation of the stochastic reaction constants and the related equations that governing the deterministic model; the model checking of the stochastic computational model; and the asymptotic solution. Click here for file

## References

[B1] Benjamins R Scheres B Auxin: the looping star in plant development Annu Rev Plant Biol 2008 59 443 465 10.1146/annurev.arplant.58.032806.103805 18444904

[B2] Kramer EM PIN and AUX/LAX proteins: their role in auxin accumulation Trends Plant Sci 2004 9 12 578 582 10.1016/j.tplants.2004.10.010 15564124

[B3] de Reuille PB Bohn-Courseau I Ljung K Morin H Carraro N Godin C Traas J Computer simulations reveal properties of the cell-cell signaling network at the shoot apex in Arabidopsis P Natl Acad Sci USA 2006 103 5 1627 1632 10.1073/pnas.0510130103 PMC1360567 16432202

[B4] Heisler MG Jönsson H Modeling auxin transport and plant development J Plant Growth Regul 2006 25 4 302 312 10.1007/s00344-006-0066-x

[B5] Jönsson H Heisler MG Shapiro BE Meyerowitz EM Mjolsness E An auxin-driven polarized transport model for phyllotaxis P Natl Acad Sci USA 2006 103 5 1633 1638 10.1073/pnas.0509839103 PMC1326488 16415160

[B6] Smith RS Guyomarc'h S Mandel T Reinhardt D Kuhlemeier C Prusinkiewicz P A plausible model of phyllotaxis P Natl Acad Sci USA 2006 103 5 1301 1306 10.1073/pnas.0510457103 PMC1345713 16432192

[B7] Feugier FG Mochizuki A Iwasa Y Self-organization of the vascular system in plant leaves: Inter-dependent dynamics of auxin flux and carrier proteins J Theor Biol 2005 236 4 366 375 10.1016/j.jtbi.2005.03.017 15899502

[B8] Feugier FG Iwasa Y How canalization can make loops: A new model of reticulated leaf vascular pattern formation J Theor Biol 2006 243 2 235 244 10.1016/j.jtbi.2006.05.022 16887150

[B9] Merks RMH Peer Y Van de Inzé D Beemster GTS Canalization without flux sensors: a traveling-wave hypothesis Trends Plant Sci 2007 12 9 384 390 10.1016/j.tplants.2007.08.004 17765595

[B10] Mitchison GJ A model for vein formation in higher plants P Roy Soc Lond B Bio 1980 207 79 109 10.1098/rspb.1980.0015

[B11] Mitchison GJ Hanke DE Sheldrake AR The polar transport of auxin and vein patterns in plants Philos T Roy Soc B 1981 295 1078 461 471 10.1098/rstb.1981.0154

[B12] Rolland-Lagan AG Prusinkiewicz P Reviewing models of auxin canalization in the context of leaf vein pattern formation in Arabidopsis Plant J 2005 44 5 854 865 10.1111/j.1365-313X.2005.02581.x 16297075

[B13] Swarup R Kramer EM Perry P Knox K Leyser HMO Haseloff J Beemster GTS Bhalerao R Bennett MJ Root gravitropism requires lateral root cap and epidermal cells for transport and response to a mobile auxin signal Nat Cell Biol 2005 7 1057 1065 10.1038/ncb1316 16244669

[B14] Goldsmith MHM Goldsmith TH Martin MH Mathematical analysis of the chemosmotic polar diffusion of auxin through plant tissues P Natl Acad Sci USA 1981 78 2 976 980 10.1073/pnas.78.2.976 PMC319928 16592983

[B15] Grieneisen VA Xu J Marée AFM Hogeweg P Scheres B Auxin transport is sufficient to generate a maximum and gradient guiding root growth Nature 2007 449 7165 1008 1013 10.1038/nature06215 17960234

[B16] Kramer EM Bennett MJ Auxin transport: a field in flux Trends Plant Sci 2006 11 8 382 386 10.1016/j.tplants.2006.06.002 16839804

[B17] Rolland-Lagan AG Encyclopedia of Life Sciences Chichester: John Wiley & Sons 2009 chap. Modelling of plant growth and development

[B18] Chavarría-Krauser A Ptashnyk M Homogenization of long-range auxin transport in plant tissues Nonlinear Anal - Real 2009 in press

[B19] Newell AC Shipman PD Sun Z Phyllotaxis: cooperation and competition between mechanical and biochemical processes J Theor Biol 2008 251 3 421 439 10.1016/j.jtbi.2007.11.036 18207165

[B20] Mitchison GJ The dynamics of auxin transport P Roy Soc Lond B Bio 1980 209 1177 489 511 10.1098/rspb.1980.0109

[B21] Keener J Sneyd J Mathematical Physiology 2004 Springer, USA

[B22] Murray JD Mathematical Biology 1989 Springer-Verlag, Berlin Heidelberg

[B23] Fisher J Henzinger TA Executable cell biology Nat Biotechnol 2007 25 11 1239 1249 10.1038/nbt1356 17989686

[B24] Shnerb NM Louzoun Y Bettelheim E Solomon S The importance of being discrete: life always wins on the surface P Natl Acad Sci USA 2000 97 19 10322 10324 10.1073/pnas.180263697 PMC27022 10962027

[B25] McCready CC Translocation of growth regulators Annu Rev Plant Physio 1966 17 283 294 10.1146/annurev.pp.17.060166.001435

[B26] Goldsmith MHM The polar transport of auxin Annu Rev Plant Physiol 1977 28 439 478 10.1146/annurev.pp.28.060177.002255

[B27] Lewis DR Muday GK Measurement of auxin transport in Arabidopsis thaliana Nat Protoc 2009 4 4 437 451 10.1038/nprot.2009.1 19282849

[B28] Romero-Campero FJ Twycross J Camara M Bennett M Gheorghe M Krasnogor N Modular assembly of cell systems biology models using P systems Int J Found Comput S 2009 20 3 427 442 10.1142/S0129054109006668

[B29] Infobiotics website 2009 http://www.infobiotic.org/

[B30] Cao Y Hall A Li H Petzold L StochKit, a new stochastic simulation toolkit Sixth International Conference on Systems Biology, Boston, M.A 2005

[B31] Spicher A Michel O Cieslak M Giavitto JL Prusinkiewicz P Stochastic P systems and the simulation of biochemical processes with dynamic compartments Biosystems 2008 91 3 458 472 10.1016/j.biosystems.2006.12.009 17728055

[B32] Hill AD Tomshine JR Weeding EMB Sotiropoulos V Kaznessis YN SynBioSS: the synthetic biology modeling suite Bioinformatics 2008 24 21 2551 2553 10.1093/bioinformatics/btn468 18757873

[B33] Sedwards S Mazza T Cyto-Sim: A formal language model and stochastic simulator of membrane-enclosed biochemical processes Bioinformatics 2007 23 20 2800 2802 10.1093/bioinformatics/btm416 17855418

[B34] Alon U Network motifs: theory and experimental approaches Nat Rev Genet 2007 8 6 450 461 10.1038/nrg2102 17510665

[B35] Priami C Algorithmic systems biology Commun ACM 2009 52 5 80 88 10.1145/1506409.1506427

[B36] Steggles LJ Banks R Shaw O Wipat A Qualitatively modelling and analysing genetic regulatory networks: a Petri net approach Bioinformatics 2007 23 3 336 343 10.1093/bioinformatics/btl596 17121774

[B37] Kaleta C Richter S Dittrich P Using chemical organization theory for model-checking Bioinformatics 2009 25 15 1915 1922 10.1093/bioinformatics/btp332 19468053 PMC2712341

[B38] Monteiro PT Ropers D Mateescu R Freitas AT de Jong H Temporal logic patterns for querying dynamic models of cellular interaction networks Bioinformatics 2008 24 16 i227 233 10.1093/bioinformatics/btn275 18689830

[B39] Batt G Ropers D de Jong H Geiselmann J Mateescu R Page M Schneider D Validation of qualitative models of genetic regulatory networks by model checking: analysis of the nutritional stress response in Escherichia coli Bioinformatics 2005 21 suppl_1 19 28 10.1093/bioinformatics/bti1048 15961457

[B40] Romero-Campero F Cao H Camara M Krasnogor N MK, et al Structure and parameter estimation for cell systems biology models Proceedings of the Genetic and Evolutionary Computation Conference (GECCO-2008) 2008 ACM Publisher 331 338 full_text

[B41] van Kampen NG Stochastic processes in physics and chemistry 1992 2 Amsterdam, The Netherlands: Elsevier Science Publishers

