# Supplementary material for: Stochastic and deterministic multiscale models for systems biology: an auxin-transport case study
Source: BMC Syst Biol. 2010 Mar 26;4:34. doi: 10.1186/1752-0509-4-34 (PMC2873313; doi:10.1186/1752-0509-4-34)
Supplement: Additional file 1 — Further model details. This document (PDF format) provides supporting information for the main text, and gives further details on the biological parameter estimates used in the model; the derivation of the stochastic reaction constants and the related equations that governing the deterministic model; the model checking of the stochastic computational model; and the asymptotic solution. [file 1752-0509-4-34-S1.PDF]

Stochastic and deterministic multiscale models for systems biology: an auxin-transport case study
Additional file 1

Jamie Twycross[1,†] , Leah R Band[1,†] , Malcolm J Bennett[1] , John R King[1,2] and Natalio Krasnogor[1,3,*]

[1]Centre for Plant Integrative Biology, School of Biosciences, Sutton Bonington Campus, University of Nottingham, Nottingham, LE12 5RD, U.K.
[2]School of Mathematical Sciences, University Park, University of Nottingham, Nottingham, NG7 2RD, U.K.
[3]Automatic Scheduling and Planning Group, School of Computer Science, Jubilee Campus, University of Nottingham, Nottingham, NG8 1BB, U.K.

Email: Jamie Twycross - jamie.twycross@cpib.ac.uk; Leah R Band - leah.band@cpib.ac.uk; Malcolm J Bennett - malcolm.bennett@nottingham.ac.uk; John R King - john.king@nottingham.ac.uk; Natalio Krasnogor*- natalio.krasnogor@nottingham.ac.uk;

*Corresponding author †Equal contributions

Introduction

These supplementary material provide supporting information for the main text. The first section describes the biological parameter estimates used in the model. In the next section, we derive the stochastic reaction constants and the related equations that governing the deterministic model. We then go on to discuss the model checking of the stochastic computational model. The final section provides further details of the asymptotic solution, and in particular derives formulae for the leading-order auxin concentrations and auxin velocity.

Biological Parameter Estimates

In this section, we discuss the biological parameter estimates required by the model. We consider a stem segment that has a length of $L = 2\,\text{mm}$, and consists of 20 cells that have a length of $l = 100\,\mu\text{m}$ and a width of $w = 10\,\mu\text{m}$. We assume that the apoplast thickness is uniform, and taken to be $\lambda = 0.5\,\mu\text{m}$ [1]. Although these dimensions are appropriate for the model species *Arabidposis thaliana*, the model could be used to investigate other plant species after a simple rescaling of the model's variables. We suppose that the source and collecting agar blocks are $L_s = 2\,\text{mm}$ long, and have a width of $10\,\mu\text{m}$. Auxin diffuses within the agar blocks, and we use the aqueous diffusion coefficient, $D = 6.7 \times 10^{-10}\,\text{m}^2\,\text{s}^{-1}$. The

remaining parameter values, pH_c, pH_w, pK, V and T are well characterised, and we use the representative values given in [1].

Key parameters in the model are the cell-membrane permeabilities, P_{diff} and P_{PIN}. Delbarre and co-workers [2, 3] measured the passive-diffusion membrane permeability in tobacco cells as $0.14 - 0.18\,\mathrm{cm\,hr^{-1}}$; therefore following previous modelling studies [1, 4], we set $P_{diff} = 0.2\,\mathrm{cm\,hr^{-1}} = 5.6 \times 10^{-7}\,\mathrm{m\,s^{-1}}$. Experimental values for the membrane permeabilities due to the efflux carriers have not been well characterised. We use $P_{PIN} = 3.3 \times 10^{-6}\,\mathrm{m\,s^{-1}}$, which is similar to the values used in previous auxin-transport studies [1, 4].

Table 1: **Transport process timescales.**

Timescale for:	Formula	Value
membrane transport the tissue length	L/P_{PIN}	140 s
membrane transport a cell length	l/P_{PIN}	7.1 s
diffusion the agar-block length	L_s^2/D	6000 s

Time scales based on the parameters values in Table 1 (given to two significant figures).

Table 1 provides estimates of the appropriate time scales for three key transport processes (using the parameter estimates in Table 1 of the main paper). These estimates suggest that membrane transport along the length of the stem segment is much faster than diffusion through the agar blocks.

Model Derivation

In this section, we derive the expressions for the reaction constants of the stochastic model ("Stochastic Computational Model" section of the main paper) and the related equations that govern the deterministic model ("Deterministic Mathematical Model" section of the main paper).

Diffusion

To model diffusion between the source agar block and the first apoplast, and the collecting agar block and the final apoplast, we use a finite-volume approximation of Fick's Law for non-uniform discretisations [5]. Thus, considering the auxin leaving the source, the flux of molecules per unit area is given by

$$J_{sa} = \frac{2D(S - a_0)}{L_s + \lambda}, \tag{1}$$

where S is the source auxin concentration, a_0 is the first-apoplast auxin concentration, L_s is the length of the source agar block, λ is the apoplast thickness and D is the diffusion coefficient.

The rate of change of number of molecules in the source is given by

$$\frac{\mathrm{d}S^n}{\mathrm{d}t} = -wJ_{sa}, \tag{2}$$

and it is straightforward to convert (3, 5-9) to a deterministic equation governing the number of molecules

$$\frac{\mathrm{d}S^n}{\mathrm{d}t} = -\frac{2D}{L_s+\lambda}\left(\frac{S^n}{L_s} - \frac{a_0}{\lambda}\right). \tag{3}$$

From (3), the stochastic reaction constants for molecules leaving and entering the source agar block are respectively given by

$$k_1 = \frac{2D}{L_s(L_s+\lambda)}, \quad k_4 = \frac{2D}{\lambda(L_s+\lambda)}, \tag{4}$$

(see Table 2 of the main paper for definitions). In addition, (3) leads readily to the deterministic equation governing the source auxin concentration ("Deterministic Mathematical Model" section of the main paper). Similarly, for the collecting agar block

$$J_{aF} = \frac{2D(a_N - F)}{L_s+\lambda}, \quad \frac{\mathrm{d}F^n}{\mathrm{d}t} = wJ_{aF}, \tag{5}$$

giving the deterministic equation governing the collecting-agar-block concentration ("Deterministic Mathematical Model" section of the main paper), and we find that the stochastic reaction constants ("Stochastic Computational Model" section of the main paper) for molecules leaving and entering the collecting agar block, k_1 and k_4 respectively, are given in (4).

Membrane Transport

Per unit length, the passive flux of protonated auxin across the cell membranes from c_i to a_i, J_{diff}, is given by (main paper Equation 3), and the passive flux from c_i to a_{i-1} is given by $P_{diff}(A_c^+ c_i - A_a^+ a_{i-1})$. In contrast, the PIN carriers facilitate anionic auxin movement across the cell membranes from c_i to a_i, and this active flux, J_{PIN}, is given by main paper Equation 3. Summing the flux components, the rate of change of the number of molecules in each cytoplasm and apoplast region is given by

$$\frac{\mathrm{d}a_0^n}{\mathrm{d}t} = w\left(\frac{2D(S-a_0)}{L_s+\lambda} + P_{diff}(A_c^+ c_1 - A_a^+ a_0)\right), \tag{6}$$

$$\frac{\mathrm{d}c_i^n}{\mathrm{d}t} = w\Big(P_{diff}(A_a^+(a_{i-1}+a_i) - 2A_c^+ c_i) + P_{PIN}(B_a a_i - B_c c_i)\Big), \tag{7}$$

$$\frac{\mathrm{d}a_j^n}{\mathrm{d}t} = w\Big(P_{diff}(A_c^+(c_j+c_{j+1}) - 2A_a^+ a_i) + P_{PIN}(B_c c_j - B_a a_j)\Big), \tag{8}$$

$$\frac{\mathrm{d}a_N^n}{\mathrm{d}t} = w\left(\frac{2D(F-a_N)}{L_s+\lambda} + P_{diff}(A_c^+ c_N - A_a^+ a_N) + P_{PIN}(B_c c_N - B_a a_N)\right), \tag{9}$$

for $i = 1, 2, \cdots, N$ and $j = 1, 2, \cdots, N-1$. It is straightforward to convert (3, 6-9) to the deterministic equations governing the auxin concentrations ("Deterministic Mathematical Model" section of the main paper).

To determine the stochastic reaction constants, we relate the flux components to the numbers of molecules, and so the rate of change of number of molecules in cytoplasm c_i due to passive diffusion is given by

$$P_{diff}\left(\frac{A_a^+(a_{i-1}^n + a_i^n)}{\lambda} - \frac{2A_c^+ c_i^n}{L}\right), \tag{10}$$

which corresponds to stochastic reaction constant

$$k_3 = \frac{A_a^+ P_{diff}}{\lambda}. \tag{11}$$

In the stochastic simulations, we assume that the small parameter $A_c^+ = 0$, so that there is no passive diffusion from the cytoplasms into the apoplast.

Similarly, the rate of change of the number of molecules in the cytoplasm, c_i^n, due to the active transport is

$$P_{PIN}\left(\frac{B_a a_i^n}{\lambda} - \frac{B_c c_i^n}{L}\right), \tag{12}$$

which gives an expression for the stochastic reaction constants ("Stochastic Computational Model" section of the main paper)

$$k_2 = \frac{B_c P_{PIN}}{L}, \quad k_5 = \frac{B_a P_{PIN}}{\lambda}. \tag{13}$$

Model Checking

We used the Prism model checker [6] to perform probabilistic model checking of the stochastic computational model. Probabilistic model checking is computationally expensive since generally the entire state space of the model needs to be calculated. The state space grows in size with the number of rules and the number of molecules. Therefore, we checked a reduced version of the computational model with a smaller state space. The reduced model uses exactly the same reactions and constants as the full model, but is reduced in length by setting $N = 2$ (i.e. considering only two cells in the cell file), giving the reduced model seven compartments in total. Since there are fewer compartments, there are also fewer rules in total. We also used an initial concentration of 1 pM ($12,000$ molecules) of auxin. The two properties that we tested are: (i) the probability that the number of molecules in the source agar block will be less than half its initial concentration; and (ii) the probability that the number of molecules in the collecting agar block will be greater than half its final concentration. The probabilities versus time are shown in Figure 3 of the main paper.

Derivation of Asymptotic Solution

Using the nondimensionalisation and scalings discussed in the "Deterministic Mathematical Model" section of the main paper, the governing equations, (6-11), become

$$\frac{\mathrm{d}S}{\mathrm{d}t} = \frac{2\epsilon L_s \bar{D}(a_0 - S)(1+\epsilon^2\bar{\lambda})}{L_s(1+\epsilon^2\bar{\lambda})+\epsilon^3\bar{\lambda}}, \tag{14}$$

$$\frac{\mathrm{d}a_0}{\mathrm{d}t} = \frac{(1+\epsilon^2\bar{\lambda})}{\epsilon^3\bar{\lambda}}\left(\frac{2\epsilon L_s^2 \bar{D}(S - a_0)(1+\epsilon^2\bar{\lambda})}{L_s(1+\epsilon^2\bar{\lambda})+\epsilon^3\bar{\lambda}} + \epsilon\bar{P}_{diff}(\epsilon\bar{A}_c^+ c_1 - A_a^+ a_0)\right), \tag{15}$$

$$\frac{\mathrm{d}c_i}{\mathrm{d}t} = \frac{(1+\epsilon^2\bar{\lambda})}{\epsilon}\left(\epsilon\bar{P}_{diff}(A_a^+(a_{i-1}+a_i) - 2\epsilon\bar{A}_c^+ c_i) + \epsilon\bar{B}_a a_i - B_c c_i\right), \tag{16}$$

$$\frac{\mathrm{d}a_j}{\mathrm{d}t} = \frac{(1+\epsilon^2\bar{\lambda})}{\epsilon^3\bar{\lambda}}\left(\epsilon\bar{P}_{diff}(\epsilon\bar{A}_c^+(c_j + c_{j+1}) - 2A_a^+ a_j) + B_c c_j - \epsilon\bar{B}_a a_j\right), \tag{17}$$

$$\frac{\mathrm{d}a_N}{\mathrm{d}t} = \frac{(1+\epsilon^2\bar{\lambda})}{\epsilon^3\bar{\lambda}}\left(\frac{2\epsilon L_s^2 \bar{D}(F - a_N)(1+\epsilon^2\bar{\lambda})}{L_s(1+\epsilon^2\bar{\lambda})+\epsilon^3\bar{\lambda}} + \epsilon\bar{P}_{diff}(\epsilon\bar{A}_c^+ c_N - A_a^+ a_N) + B_c c_N - \epsilon\bar{B}_a a_N\right), \tag{18}$$

$$\frac{\mathrm{d}F}{\mathrm{d}t} = \frac{2\epsilon L_s \bar{D}(a_N - F)(1+\epsilon^2\bar{\lambda})}{L_s(1+\epsilon^2\bar{\lambda})+\epsilon^3\bar{\lambda}}, \tag{19}$$

for $i = 1, 2, \cdots, N$, and $j = 1, 2, \cdots, N-1$, where we drop the hats for convenience.

In the stem segment, we take a continuum limit: we let x measure the length along the tissue, such that $x = \epsilon i$ (where $x = 0$ corresponds to the upper face of cytoplasm $i = 1$), and set

$$c_i(t) = \bar{c}(x,t), \quad c_{(i+1)} = \bar{c} + \epsilon\frac{\partial \bar{c}}{\partial x} + O(\epsilon^2), \tag{20}$$

$$a_i(t) = \bar{a}(x,t), \quad a_{(i-1)} = \bar{a} - \epsilon\frac{\partial \bar{a}}{\partial x} + O(\epsilon^2), \tag{21}$$

using a Taylor series approximation. We note that $a_0(t) = \bar{a}(0,t)$, $c_1(t) = \bar{c}(0,t)$, $a_{N-1}(t) = \bar{a}(1,t)$ and $c_N(t) = \bar{c}(1,t)$, and therefore solve for the five variables $S(t)$, $F(t)$, $\bar{c}(x,t)$, $\bar{a}(x,t)$ and $a_N(t)$. In addition, we expand the variables using standard perturbation series, for example, $S(t) = S_0(t) + \epsilon S_1(t) + O(\epsilon^2)$, and construct asymptotic solutions for the first non-zero term in each expansion.

On the transport time scale, $t = O(1)$, equation (14) suggests that there is no significant depletion of the source concentration at leading order, $S_0(t) = 1$, and therefore considering (15) at leading order, the leading-order concentration in the first apoplast can be simply calculated via

$$\bar{a}_0(0,t) = a_{00}(t) = \frac{2L_s\bar{D}}{2L_s\bar{D} + A_a^+\bar{P}_{diff}}. \tag{22}$$

Using (21), equations (16,17) at leading order show that the cytoplasm concentrations are $O(\epsilon)$ ($\bar{c}_0 = 0$), and that

$$\bar{c}_1(x,t) = \frac{(2A_a^+\bar{P}_{diff} + \bar{B}_a)}{B_c}\bar{a}_0(x,t). \tag{23}$$

Then proceeding to next order, we obtain the solvability condition from the summation of (16,17), and thus, the cytoplasm concentrations are governed by the wave equation

$$\frac{\partial \bar{c}_1}{\partial t} + v_{eff}\frac{\partial \bar{c}_1}{\partial x} = 0, \quad v_{eff} = \frac{A_a^+ B_c \bar{P}_{diff}}{(2A_a^+ \bar{P}_{diff} + \bar{B}_a)}. \tag{24}$$

Finally, from (19), the collecting-agar-block concentration is $O(\epsilon)$. Summing (16) with $i = N$ to (18), we find

$$\begin{aligned} a_{N0}(t) &= \frac{A_a^+ \bar{P}_{diff}}{2L_s\bar{D}} a_{(N-1)0}(t), \\ &= \frac{A_a^+ \bar{P}_{diff}}{2L_s\bar{D}} \bar{a}_0(1,t). \end{aligned} \tag{25}$$

Then equation (19) gives

$$\begin{aligned} F_1(t) &= 2\bar{D}\int_0^t a_{N0}\,\mathrm{d}t, \\ &= \frac{A_a^+ \bar{P}_{diff}}{L_s}\int_0^t \bar{a}_0(1,t)\,\mathrm{d}t, \\ &= \frac{v_{eff}}{L_s}\int_0^t \bar{c}_1(1,t)\,\mathrm{d}t. \end{aligned} \tag{26}$$

We now consider the slower time scale, $t = \tilde{t}/\epsilon$, on which the source concentration depletes. On this time scale, the leading-order equations (14, 15) provide the simple formulae

$$S_0(\tilde{t}) = \exp\left(-\frac{2\bar{D}A_a^+ \bar{P}_{diff}\tilde{t}}{2L_s\bar{D} + A_a^+ \bar{P}_{diff}}\right), \tag{27}$$

$$\bar{a}_0(0,\tilde{t}) = \frac{2L_s\bar{D}}{2L_s\bar{D} + A_a^+ \bar{P}_{diff}} S_0(\tilde{t}). \tag{28}$$

From (16,17),

$$\bar{c}_1(x,\tilde{t}) = \frac{(2A_a^+ \bar{P}_{diff} + \bar{B}_a)}{B_c}\bar{a}_0(x,\tilde{t}), \tag{29}$$

and then the summation of (16,17) shows that

$$\frac{\partial \bar{c}_1}{\partial x} = \frac{\partial \bar{a}_0}{\partial x} = 0, \tag{30}$$

Thus, on the slow time scale, the concentrations in the stem segment are in equilibrium, so that $\bar{a}_0(x,\tilde{t})$ and $\bar{c}_1(x,\tilde{t})$ are independent of x. Summing (16) with $i = N$ to (18), we find

$$\begin{aligned} F_0(\tilde{t}) - a_{N0}(\tilde{t}) &= -\frac{A_a^+ \bar{P}_{diff}}{2L_s\bar{D}}\bar{a}_0(1,\tilde{t}) \\ &= -\frac{A_a^+ \bar{P}_{diff}}{2L_s\bar{D} + A_a^+ \bar{P}_{diff}} S_0(\tilde{t}), \end{aligned} \tag{31}$$

using (28). Then substituting (31) into the leading-order equation governing the collecting-agar-block concentration, (19), we find

$$F_0(\tilde{t}) = 1 - \exp\bigg(- \frac{2\bar{D}A_a^+\bar{P}_{diff}\tilde{t}}{2L_s\bar{D} + A_a^+\bar{P}_{diff}}\bigg). \tag{32}$$

To summarise, on the time scale of membrane transport the auxin concentration in the source agar block is approximately constant and auxin travels through the stem segment with a defined front according to (24). This equation, (24), provides a formula for the speed of auxin transport through the stem segment. On redimensionalising, we find

$$\text{Auxin speed} = \frac{A_a^+P_{diff}B_cP_{PIN}}{(2A_a^+P_{diff} + B_aP_{PIN})} \approx 1.95\,\text{cm}\,\text{hr}^{-1}, \tag{33}$$

and the time at which significant auxin first enters the collecting agar block is given by

$$t = \frac{L(2A_a^+P_{diff} + B_aP_{PIN})}{A_a^+P_{diff}B_cP_{PIN}} \approx 356.91\,\text{seconds}, \tag{34}$$

in terms of the dimensional parameter estimates in Table 1 of the main paper. At early times, negligible auxin enters the collecting agar block; once the wave of auxin has travelled through the stem segment, the auxin concentration in the final cytoplasm is constant and therefore the collecting-agar-block concentration increases linearly with time according to

$$F_1(t) = \frac{2\bar{D}A_a^+\bar{P}_{diff}}{2L_s\bar{D} + A_a^+\bar{P}_{diff}}\bigg(t - \frac{1}{v_{eff}}\bigg), \tag{35}$$

from (22, 23, 26, 34). Thus, by measuring the time at which significant auxin first enters the collecting agar block (denoted t_e), the effective velocity can be calculated via $v_{eff} = L/t_e$. In the "Execution Times" section of the main paper, we discuss the number of molecules in the collecting agar block at 723.78 seconds; this is equivalent to $t = 1.19$, and gives $F \sim \epsilon F_1 = 0.0204$.

On a slower time scale, the auxin concentrations in the stem segment are uniform throughout the tissue (*i.e.* do not depend on the distance from the source agar block). When $\tilde{t}$ is small, the concentration in the collecting agar block, (32), increases linearly with time, according to

$$F_0(\tilde{t}) = \frac{2\bar{D}A_a^+\bar{P}_{diff}}{2L_s\bar{D} + A_a^+\bar{P}_{diff}}\tilde{t}, \tag{36}$$

which matches the fast-time-scale solution, (35), as $t \to \infty$. In the "Auxin Concentrations"section of the main paper, we consider the time at which the agar-block concentrations are half their equilibrium value; the asymptotic solution, (27, 32), suggests that this time is approximately the same for both the source

and the sink agar blocks, and is given by

$$\tilde{t} = \frac{(2L_s\bar{D} + A_a^+\bar{P}_{diff})\ln(2)}{2\bar{D}A_a^+\bar{P}_{diff}}, \tag{37}$$

which in dimensional variables gives

$$t = \frac{L_sL(2D + L_sA_a^+P_{diff})\ln(2)}{2DLA_a^+P_{diff}} \approx 206.21\,\text{min}. \tag{38}$$

On long time scales, the auxin concentration in the collecting agar block, (32), cannot be used to calculate the auxin velocity through the stem segment, (33). As discussed in [7, 8], this experimental protocol has been used to consider both the distance moved per unit time (the velocity) and the amount of auxin passing through the tissue per unit time (the flux). Here we have shown that on long time scales diffusion within the agar blocks determines the rate at which auxin enters the collecting agar block, and therefore may affect the quoted auxin velocities and fluxes. To measure the auxin velocity unambiguously we need to look at the time at which the first auxin molecules typically enter the collecting agar block: although we can do this in our modelling framework, it is likely to be impractical to measure this time experimentally. These conclusions are valid only where the lengths of agar blocks are comparable to the length of the stem segment; the effect of diffusion within the agar blocks will be much smaller with smaller agar blocks.

References

1. Swarup R, Kramer EM, Perry P, Knox K, Leyser HMO, Haseloff J, Beemster GTS, Bhalerao R, Bennett MJ: **Root gravitropism requires lateral root cap and epidermal cells for transport and response to a mobile auxin signal**. *Nat. Cell Biol.* 2005, **7**:1057–1065.
2. Delbarre A, Muller P, Imhoff V, Guern J: **Comparison of mechanisms controlling uptake and accumulation of 2, 4-dichlorophenoxy acetic acid, naphthalene-1-acetic acid, and indole-3-acetic acid in suspension-cultured tobacco cells**. *Planta* 1996, **198**(4):532–541.
3. Delbarre A, Muller P, Imhoff V, Morgat JL, Barbier-Brygoo H: **Uptake, accumulation and metabolism of auxins in tobacco leaf protoplasts**. *Planta* 1994, **195**(2):159–167.
4. Heisler MG, Jönsson H: **Modeling auxin transport and plant development**. *J. Plant Growth Regul.* 2006, **25**(4):302–312.
5. Bernstein D: **Simulating mesoscopic reaction-diffusion systems using the Gillespie algorithm.** *Phys. Rev. E* 2005, **71**:041103.
6. Hinton A, Kwiatkowska M, Norman G, Parker D: **PRISM: a tool for automatic verification of probabilistic systems**. In *Proc. of the 12th International Conference on Tools and Algorithms for the Construction and Analysis of Systems*, *Volume 3920 of* LNCS. Edited by Hermanns H, Palsberg J, Springer 2006:441–444.
7. McCready CC: **Translocation of growth regulators**. *Annu. Rev. Plant Physio.* 1966, **17**:283–294.
8. Lewis DR, Muday GK: **Measurement of auxin transport in Arabidopsis thaliana**. *Nat. Protoc.* 2009, **4**(4):437–451.
